# Building long-term vision for rural areas through multi-actor platforms: a preliminary study in the Emilia-Romagna region

**DOI:** 10.12688/openreseurope.13293.1

**Published:** 2021-04-21

**Authors:** Emilia Pellegrini, Meri Raggi, Davide Viaggi, Stefano Targetti

**Affiliations:** 1 Department of Agricultural Sciences, University of Bologna, Bologna, 40127, Italy; 2 Department of Statistical Sciences, University of Bologna, Bologna, 40126, Italy

**Keywords:** Desirable future; Backcasting; stakeholders’ consultation; evidence-based; multi-actor approach

## Abstract

Developing long-term visions through participatory approaches can be very useful to explore different possible scenarios and pathways to reach desirable futures. This brief report describes a participatory process carried out in the Emilia-Romagna region (Italy) to develop a long-term vision for rural areas by 2040. This approach consisted of: (i) interviews and a focus group carried out with a multi-actor platform (MAP) composed of experts from science-society-policy sectors, and (ii) an on-line questionnaire addressing a larger number of rural stakeholders of the region. Mixing expert-based consultation through the MAP with a more inclusive consultation approach resulted in an effective method to build long-term visions in the very heterogeneous rural context of the Emilia-Romagna. However, this study only constitutes a preliminary step into a more elaborated backcasting approach.

## Plain language summary

How do we imagine rural areas of the future? What obstacles should be removed to achieve a desirable future and to empower rural areas and their inhabitants? We tried to answer these questions by engaging rural stakeholders of the Emilia-Romagna region (Italy) in developing their own long-term vision for rural areas by 2040. This report presents the method and the results of the participatory process carried out in the region that led to the creation of two long-term visions for rural areas.

## Introduction

The current health and economic crises, originating from the coronavirus disease 2019 (COVID-19) pandemic, have caused several challenges and opportunities for the future of European rural areas. On one hand, the gap among urban and rural areas has been exacerbated by the crisis and it is likely to be amplified in the coming years ( OECD, 2020). On the other hand, lifestyles and consumption patterns have undergone deep transformations that are creating new opportunities for rural areas. Many policy documents have stressed the urgency to seize these opportunities to make rural areas the heart of a strategy for a climate neutral Europe, and to make them benefit from a great digital acceleration ^1^. It is in this moment of rethinking the roles and opportunities for rural areas that the European Commission (EC) launched a process of public consultation to develop a long-term vision for rural areas with a time horizon of 2040 with the aim of “gathering a bottom-up evidence base […] on the needs and aspirations of European citizens” ( European Commission, 2020).

Visions are often used in sustainability research when complex human-environment problems are concerned, the future is uncertain, and a sense of dissatisfaction with the current state is diffused ( Meadows & O’Brien, 2006; Staricco *et al.*, 2019). Developing a vision usually constitutes the first step in backcasting. In contrast to forecasting studies that explore the most likely projections of the future, backcasting is a normative approach to identify desirable futures and their implications ( Robinson, 2003). Working on visions usually requires multi-actor approaches to bring different expertise and interests into the process. Think-tanks including experts and researchers ( Staricco *et al.*, 2019), Delphi surveys with a large pool of experts ( Zimmermann *et al.*, 2012), and workshops with stakeholders ( Sisto *et al.*, 2017) are some of the most common methods to engage actors into the development of visions.

This brief report describes the multi-actor approach developed for the elaboration of a long-term vision for rural areas of the Emilia-Romagna region (Italy). The study is part of a larger consultation process carried out within the Horizon 2020 project SHERPA (Sustainable Hub to Engage into Rural Policies with Actors) aimed at creating multi-actor platforms (MAPs) at member states and EU level, to improve dialogue between local rural territories and EU institutions. Given the synergies between SHERPA’s objectives and the EC consultation on long-term vision, the MAPs have worked to create their own visions for rural areas in 2040 since spring 2020. The stakeholder-based information gathered through the MAPs fed into a SHERPA position paper ( Chartier *et al.*, 2021) aimed to create a channel of communication among rural territories and the EC.

The following sections describe the process undertaken within the Emilia-Romagna MAP to create a long-term vision for rural areas of the region.

## Methods

This study complies with EU GDPR Regulation and written informed consent was obtained from the participants.

### Study area

Emilia-Romagna is a region (NUTS2) of the north-east of Italy characterized by very heterogeneous rural areas, ranging from hilly-mountainous areas located along the Apennines ridge, to rural areas located in the Po plain. Characteristics of hilly-mountainous areas are, on one hand, unique environments, extensive agriculture and strong cultural and historical identity. On the other hand, these areas are affected by negative demographic trends, land-abandonment and hydrogeological instability. Rural areas of the plain are characterized by intensive and competitive agriculture, farm concentration, homogenization of agricultural landscape structure.

### Setting up the Emilia-Romagna MAP

A MAP is an arrangement composed of three societal groups (researchers, policy-makers and society) that stimulates dialogue, engagement and co-construction of knowledge regarding EU rural policy and research agenda ( Nordregio, 2020). The first principle guiding the composition of the Emilia-Romagna MAP was to have at least one representative from science, society and policy. A second criterion was to have actors representing both types of rural areas of the region. Lastly, experts with a cross-sectoral expertise on agricultural and rural development were preferred to avoid excessively biased opinions. Eventually, 13 experts were invited to take part to the development of the vision, but only seven replied to the invitation. The experts allowed a good coverage of all types of rural areas of the region. On the other hand, the policy sector was under-represented (MAP composition: two science; four society; one policy).

### Development of long-term vision for rural areas

Stakeholders’ consultation for long-term vision was based on three techniques: interviews, a focus group, and an on-line questionnaire. Interviews and the focus group were conducted with the MAP and can be described as an expert-based consultation that allowed to draft a first version of the vision. The questionnaire, instead, aimed at involving a larger number of stakeholders in the visioning exercise. More information on each technique is provided below.

***Interviews.*** Seven on-line semi-structured interviews were conducted with MAP’s members aimed at identifying: (i) the main challenges and opportunities for rural areas of the region from now to 2040; (ii) a vision for rural areas in 2040; (iii) obstacles to achieving the vision; (iv) enablers to achieve the vision. In the analysis of interviews, the arguments raised by the MAP’s experts were organized around two visions, one for hilly-mountainous rural areas and another for rural areas of the plain.

***Focus group.*** The two visions emerged from the interviews were presented to the MAP during an on-line focus group. The meeting was attended by five members of the MAP (one policy; four society) plus three researchers that facilitated the meeting. Participants were invited to give feedback on the visions and on barriers and enablers for their achievement. The discussion led to a reformulation of the vision for hilly-mountainous areas and the new version was sent by e-mail to all the MAP’s members for additional comments.

***Questionnaire.*** Qualitative data acquired through the interviews and focus group were used to develop a questionnaire ( Pellegrini *et al.*, 2021) to be circulated to 87 stakeholders selected for their expertise in the agri-food sector and rural development. Both visions were reformulated in terms of improvements for regional rural areas in 2040: notably, seven possible improvements were identified for hilly-mountainous areas, while four improvements for rural areas of the plain. Following Zimmermann *et al.* (2012), for each improvement respondents were asked to rate its desirability on a five point Likert-scale and the probability for it to occur in 2040 (0–100%). Furthermore, participants were invited to provide qualitative arguments related to obstacles for the achievement of desired changes. Arguments were analysed through an inductive coding with the scientific software Atlas.ti 8 that supported the identification of recurring themes.

***Validation of the visions.*** Data collected through the questionnaire were used to reframe and fine-tune both visions. The updated version was sent by email to the MAP for validation.

## Results

Both visions presented in this section include the results collected within the MAP and through the on-line questionnaire. The latter was completed by 23 stakeholders (26,4% response rate) the majority of which had a professional background in business/industry (46%) followed by public administration (33%) (research 8%; Other 8%; NGO/civil society 4%).

### A vision for hilly mountainous rural areas

In 2040, infrastructures will be improved in hilly-mountainous areas. This will facilitate mobility of people and goods within the region and the creation of new services in rural areas. The damages related to hydrogeological instability will be prevented and contained thanks to the enhancement of infrastructures.

Rural areas will be more connected to the global world due to an increase in digital infrastructures. Digitalization will be based on local needs and will lead to the creation of new job opportunities.

The rural economy will be sustained by a thriving agriculture based on the valorisation of typical agri-food and wine productions and on the enhancement and remuneration of ecosystem services linked to agriculture. Non-agricultural activities, such as tourism, will also contribute to the viability of rural areas. Rural areas will become the place where a range of services linked to nature, culture and wellbeing will be available to the rural and urban populations.

**Figure 1.**
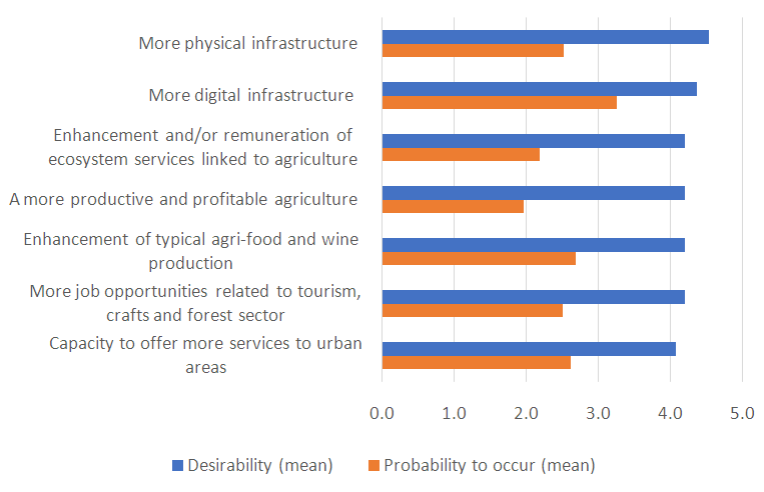
Mean values of desirability and probability to occur (on a 5-point scale) of improvements for hilly-mountainous rural areas of Emilia-Romagna. To ease the readability of the graph, the probability was also represented on a 5-point scale.

All the improvements identified by the MAP were considered very desirable by respondents to the questionnaire ( Figure 1). However, some changes were considered less probable to occur in 2040 compared to others. To understand the gap between desirability and feasibility of improvements, we analysed the arguments raised by stakeholders on obstacles to desired changes. For brevity, here we report only on the three improvements for which the gap between desirability and feasibility was higher. Barriers to the enhancement of physical infrastructures (roads, bridges, etc.) were identified in the lack of adequate investments and in political inability for long-term programming. Obstacles to a more productive and profitable agriculture were identified in the strong competition created by global markets and in their large-scale distribution systems. These, indeed, penalize local agri-food production characterized by niche dimensions due to the territorial conformation and the wide fragmentation of farm structure. Lastly, cultural barriers and lack of awareness were cited as main barriers for the enhancement and valorisation of ecosystem services.

### A vision for rural areas of the plain

In 2040, rural areas of the plain will be more resilient to climate change. Infrastructures, such as flood retention basins, will be key to enhance resiliency of these areas but a greater economic support to farms and a strengthening of risk management and compensation tools will be also needed. The collaboration among farms in the management of water resources, for example through the creation of collective reservoirs, will be strengthened to face water-related problems. The organization of the supply chain will be improved with a strengthening of the cooperative system. This would allow for the greater participation of farmers in the supply chain and will sustain the consumption of local agri-food products.

**Figure 2.**
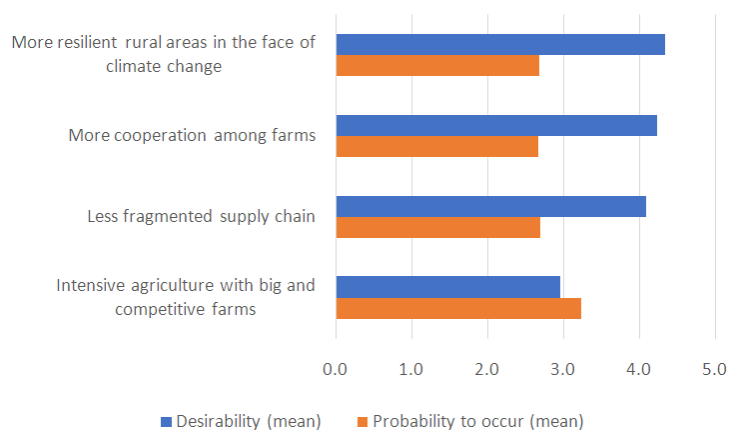
Mean values of desirability and probability to occur (on a 5-point scale) of improvements for rural areas of the plain of Emilia-Romagna. To ease the readability of the graph, the probability was also represented on a 5-point scale.

The improvements identified by the MAP were considered very desirable by the respondents to the questionnaire except for “intensive and competitive agriculture dominated by big players” that in fact was not included in the vision. On the opposite, this change was deemed the most likely to occur in 2040 ( Figure 2) because it is more aligned with the current situation. Ensuring the competitiveness of agriculture in the future was highlighted as a priority by the MAP. However, the arguments raised both by the MAP and by respondents to the questionnaire stress the importance of boosting a competitive agriculture based on quality production, both in terms of products and processes, rather than quantity. In this regard, the competitiveness of global markets, that do not valorise the quality of production, was again considered a barrier.

The resistance of farmers to collaborate among each other was mentioned as an obstacle for the achievement of all the other desired changes. Lastly, the incentives that should enable a better organization of supply chain were deemed not appropriate by some respondents.

## Conclusions

This brief report describes the multi-actor approach developed to elaborate a long-term vision for rural areas of the Emilia-Romagna region.

The approach employed different methods for stakeholders’ consultation to gather both qualitative and quantitative information; furthermore, expert-based consultation within a MAP was associated with a survey addressing a larger number of stakeholders, in order to bring more perspectives into the visions. Mixing different methods was found to be effective given the breadth of the topic and the complexity and heterogeneity characterising rural areas of the region. On one hand, having a group of core experts helped to set the boundaries of discussion and to identify the most relevant themes for rural areas. On the other hand, data collected through the questionnaire allowed for a prioritization of topics and for setting the basis for employing a backcasting approach that is fundamental to translate the visions into concrete actions.

However, the limited number of stakeholders involved makes this study only a preliminary step into a more robust process of vision development; moreover, the inputs collected remain very general. To gain more grounded results, future studies may consider testing both visions against a set of explorative scenarios to discuss with stakeholders the concrete implications for their achievement.

## Data availability

### Underlying data

AMS Acta: Dataset related to the brief report "Building long-term vision for rural areas through Multi-actor Platform: a preliminary study in the Emilia-Romagna region"

http://doi.org/10.6092/unibo/amsacta/6612 ( Pellegrini *et al.*, 2021)

This project contains the following underlying data:

- Dataset_Long-term vision for rural areas of Emilia-Romagna. The Dataset contains: 1 Excel file providing the results of the on-line questionnaire; 1 PDF file containing a blank version of the on-line questionnaire; 1 README file including a description of the Dataset. More information regarding the outputs and analysis of the interviews and the focus group are available at: https://rural-interfaces.eu/wp-content/uploads/2021/02/MAP_Position-Paper_IT_Emilia-Romagna_LTVRA.pdf

Data are available under the terms of the Creative Commons Attribution 4.0 International license (CC-BY 4.0).

### Extended data

AMS Acta: Dataset related to the brief report "Building long-term vision for rural areas through Multi-actor Platform: a preliminary study in the Emilia-Romagna region"

http://doi.org/10.6092/unibo/amsacta/6612 ( Pellegrini *et al.*, 2021)

This project contains the following extended data:

- A blank version of the on-line questionnaire is provided in the Dataset as extended data. Note: this brief report is built exclusively on answers related to the questions on the visioning exercise; however, the on-line questionnaire includes several additional questions whose answers were not included in this study.

Data are available under the terms of the Creative Commons Attribution 4.0 International license (CC-BY 4.0).
