# Supplementary material for: Building long-term vision for rural areas through multi-actor platforms: a preliminary study in the Emilia-Romagna region
Source: Open Res Eur. 2021 May 26;1:41. Originally published 2021 Apr 21. [Version 2] doi: 10.12688/openreseurope.13293.2 (PMC10445850; doi:10.12688/openreseurope.13293.2)
Supplement: Peer review history

# Peer review: Reviewer response for version 2

**DOI:** 10.21956/openreseurope.14805.r26986

**Published:** 2021-05-27

**Authors:** Paweł Chmieliński

**Affiliations:** 1 Institute of Rural and Agricultural Development of the Polish Academy of Sciences, Warsaw, Poland

The improved version of the article fully meets the requirements of a 'Brief Report' as defined by Open Research Europe and is definitely worth indexing.

Are all the source data and materials underlying the results available?

Yes

Is the study design appropriate and is the work technically sound?

Yes

Is the work clearly and accurately presented and does it engage with the current literature?

Yes

Are the conclusions drawn adequately supported by the results?

Yes

Are sufficient details of methods and analysis provided to allow replication by others?

Yes

Reviewer Expertise:

Rural systems, community-led development, family farms, institutional economics, public policy.

I confirm that I have read this submission and believe that I have an appropriate level of expertise to confirm that it is of an acceptable scientific standard.

# Peer review: Reviewer response for version 1

**DOI:** 10.21956/openreseurope.14363.r26789

**Published:** 2021-05-21

**Authors:** Paweł Chmieliński

**Affiliations:** 1 Institute of Rural and Agricultural Development of the Polish Academy of Sciences, Warsaw, Poland

A very good, brief study report showing a method of bottom-up work within a multi-actor group of local stakeholders to obtain information about regional challenges, opportunities and long-term vision in a systematic and structured way, which can be used in the preparation of a broader framework for public policy programming. 

 The results described justify and support the different research techniques: interviews, a focus group, and an online questionnaire used in practice, and allow the formulation of valuable conclusions. The study as a whole, together with the references to broader sources that provide more information on the research techniques of the study, can be regarded as a good reference for studies of a similar nature. This constitutes the universal value of the material presented.

Are all the source data and materials underlying the results available?

Yes

Is the study design appropriate and is the work technically sound?

Yes

Is the work clearly and accurately presented and does it engage with the current literature?

Yes

Are the conclusions drawn adequately supported by the results?

Yes

Are sufficient details of methods and analysis provided to allow replication by others?

Yes

Reviewer Expertise:

Rural systems, community-led development, family farms, institutional economics, public policy.

I confirm that I have read this submission and believe that I have an appropriate level of expertise to confirm that it is of an acceptable scientific standard.

# Response

**Published:** 2021-05-24

**Authors:** Emilia Pellegrini

**Affiliations:** University of Bologna, Italy

Dear Reviewer, thank you very much for your valuable comments. One of the purposes of this brief report was, indeed, to provide a concise description of the method applied to develop long-term visions in contexts characterized by high heterogeneity. We found that mixing different types of techniques, along with the decision of differentiating the visions according to the type of rural areas, were appropriate methodological choices to organize information on such a broad topic in a way that could be informative for policy documents and regional programming. Kind regards

# Peer review: Reviewer response for version 1

**DOI:** 10.21956/openreseurope.14363.r26790

**Published:** 2021-05-17

**Authors:** Emil Erjavec

**Affiliations:** 1 Biotechnical Faculty, University of Ljubljana, Ljubljana, Slovenia

The paper is a brief report presenting the results of a participatory process carried out in the Emilia-Romagna region (Italy) to develop a long-term vision for rural areas by 2040. These rural areas are changing due to both long-term and current economic and environmental patterns. The gap between urban and rural areas is not closing but is actually worsening. The discussion of the vision can help to determine the decisive factors for targeted societal changes and to elaborate the appropriate public and private policy mix. The authors’ methodology consisted of interviews, focus group and an on-line questionnaire applied within a multi-actor platform (MAP) composed of experts representing different institutions from science, society and policy.

 Due to the region’s economic and natural characteristics, two visions were developed – one for hilly-mountainous areas and one for flatland (plain) rural areas. The vision for hilly-mountainous rural is driven by two main elements: the development of infrastructure and market valorisation of typical agro-food products based on ecosystem services and promoted through tourism. The vision for the plain areas deals mainly with the strengthening of farm and village resilience to climate change, focusing on water management and farm risk management.  

 Mixing expert-based consultation through the MAP with an on-line survey approach resulted in a simple and effective method to build long-term visions in this segment of rural areas within the very heterogeneous frame of rural areas in Emilia-Romagna.

 The short paper is adequately prepared and well written. The paper can be seen as an example of a research-based alternative for the elaboration of "visions" as strategic papers in the public domain. This is the main value-added part of this paper in addition to the substantive results, which show the importance of making a clear distinction between different parts of rural territories. We can agree with the statement of the authors that this brief study “only constitutes a preliminary step into a more elaborated backcasting approach”. The sample is small, the research questions are simplified, narrowed to the elaboration of key priorities and direction of future public and private intervention. However, this type of research is useful and can contribute to the formation of better strategic public documents; I therefore recommend that the paper be indexed.

Are all the source data and materials underlying the results available?

Yes

Is the study design appropriate and is the work technically sound?

Yes

Is the work clearly and accurately presented and does it engage with the current literature?

Yes

Are the conclusions drawn adequately supported by the results?

Yes

Are sufficient details of methods and analysis provided to allow replication by others?

Yes

Reviewer Expertise:

Agricultural policy, European integration, Political economy of agriculture.

I confirm that I have read this submission and believe that I have an appropriate level of expertise to confirm that it is of an acceptable scientific standard.

# Response

**Published:** 2021-05-19

**Authors:** Emilia Pellegrini

**Affiliations:** University of Bologna, Italy

Dear Reviewer, thank you very much for your comments. We are happy to know that you believe this report can contribute to the formation of strategic public documents even though, of course, it was only a preliminary study. We hope, in fact, that this study can provide easily readable information to all those who are interested in rural areas and in applied methods to develop long-term visions.  Kind regards

# Peer review: Reviewer response for version 1

**DOI:** 10.21956/openreseurope.14363.r26786

**Published:** 2021-05-11

**Authors:** Paolo Prosperi

**Affiliations:** 1 Mediterranean Agronomic Institute of Montpellier, Montpellier, France

Dear Editors,

 Thank you for giving me the opportunity to provide a peer-review of this Brief Report.

 The brief report accurately synthetises the methodological effort that authors did to envision a desirable future for rural areas in the Italian region of Emilia-Romagna, through participatory and transdisciplinary research.

 Authors are able to gather science-policy-society actors to formulate, validate, fine-tuning and testing visions for rural areas through three main methodological steps, namely expert interviews, focus group, and an on-line broad questionnaire. 

 Findings mainly highlight the gap between desirable actions and realistic outcomes that can be reached according to both potential and obstacles that might occur.

 The report clearly describes the scientific and methodological approach within the context of a European Horizon 2020 project. The work and its scope are introduced adequately, as well as the methodological approach is properly defined. Authors acknowledge that results are still partial as the sample of experts involved is not enough large and future studies will need larger actors involvement. 

 Few minor comments:

- While scientific and specialised audience knows what a backcasting technique is, a reader might not interpret correctly such term in this context. I would suggest to add no more than one or two lines to briefly define "backcasting" in the second short paragraph of the introductory section.
- I am not sure that "evidence-based" has its place in the keywords as it is an adjective (even if it is a composed one). This might be replaced maybe by "evidence base".
- In the conclusion I would suggest to add, in the end, few information about the next steps for this MAP exercise.

Are all the source data and materials underlying the results available?

Yes

Is the study design appropriate and is the work technically sound?

Yes

Is the work clearly and accurately presented and does it engage with the current literature?

Yes

Are the conclusions drawn adequately supported by the results?

Yes

Are sufficient details of methods and analysis provided to allow replication by others?

Yes

Reviewer Expertise:

Agricultural and food economics, food policy, sustainable food systems.

I confirm that I have read this submission and believe that I have an appropriate level of expertise to confirm that it is of an acceptable scientific standard.

# Response

**Published:** 2021-05-14

**Authors:** Emilia Pellegrini

**Affiliations:** University of Bologna, Italy

Dear Reviewer, thank you very much for your accurate revision and comments. We have addressed all the observations that you made in the revised version of the brief report. Notably, we have included a brief explanation of the backcasting approach in the introduction. Moreover, in the conclusion, we have outlined the future activities of the Emilia-Romagna MAP. We could not address your comment on the keywords, instead, because keywords were not editable.  Kind regards, Emilia

# Peer review: Reviewer response for version 1

**DOI:** 10.21956/openreseurope.14363.r26788

**Published:** 2021-05-07

**Authors:** Isabel Bardaji, Carina Folkeson-Lillo

**Affiliations:** 1 Research Centre for the Management of Agricultural and Environmental Risks (CEIGRAM), Universidad Politécnica de Madrid, Madrid, Spain

**REVIEW**

 Brief report:

 Building long-term vision for rural areas through multi-actor platforms: a preliminary study in the Emilia-Romagna region

 Authors: Emilia Pellegrini, Meri Raggi, Davide Viaggi, Stefano Targetti

 Reviewers: Isabel Bardají and Carina Folkeson-Lillo. CEIGRAM, Universidad Politécnica de Madrid

**Minor observations **

1. In the introduction the authors point that “On one hand, the gap among urban and rural areas has been exacerbated by the crisis and it is likely to be amplified in the coming years (OECD, 2020)”. – what gap? Which gap is referred to? Economic? Social? Could be better specified. 
2. They say also than “On the other hand, lifestyles and consumption patterns have undergone deep transformations that are creating new opportunities for rural areas”. Our opinion is that the wording "deep transformations" is exaggerated. We have no proof for this yet. We only know that during this year, online shopping has increased (but not always that drastically), and that some people have taken the decision to move to the countryside (but a rather limited share of the society, and we don't know if this is a permanent move or only temporary). 
3. Another reflection in relation to the analysis of the results obtained concern the potential differences of opinions which may have been captured during the consultation process. Hence, were the differences of opinions between various experts very significant, or was there a general consensus among the experts? And were there great differences of opinions between the experts consulted and the survey respondents? These aspects could be further reflected upon in the paper, as this may provide further insights into the likeliness of obtaining a certain desired outcome. The greater the consensus, the likelier that a desired outcome will also be a probable outcome.

**General comments**

 The brief report raises a timely and relevant topic for the coming EC Communication on the Long-term Vision for rural areas in the 2040 horizon. It focuses on a region, Emilia-Romagna, which has two very different areas with diverse endowments and potential development. A mountainous area, with a high environmental and cultural resources affected by depopulation problems, and another area located in the Po valley with a very competitive agriculture. This diversity enriches the analysis and conclusions.

 The methodology used, based on direct semi-structured interviews and focus groups carried out on multi-actor platforms and surveys to selected stakeholder, is well planned and is adequate for the objectives pursued. A reflection about the limitations of participatory methodologies comes out and its use in situations imposed by the current pandemic situation caused by Covid-19 and the need to resort to online meetings. Meetings characterized by less agile debates and greater constraints in oral communication and transmission of ideas and knowledge. To what extent this may have an effect on participation and results obtained is an issue not studied so far and that should receive future attention by the scientific community in the near future in order to improve the use of these methodologies in a confinement situation.

 Another reflection arises from the analysis of the results obtained. It has already been mentioned that the long-term vision of two areas with very different features and therefore with different development possibilities is addressed. It is not surprising that the vision of both areas has its own characteristics. However, it is possible to extract common aspects that denote its importance for the future of the rural world: the improvement of infrastructures and the better integration of the agricultural sector in the value chain.

 The deficiencies in the provision of infrastructure, social, physical or economic, constitute an important restriction for rural development and its improvement is a necessary, although not sufficient, condition for this to occur. It is not surprising, therefore, that its improvement is included in both cases as a desirable aspect in that long-term vision and that issues such as improving mobility or the creation of new services in the most remote areas or control of flooding in the valley area are included.

 Improving the value chain of agricultural and food production has long been a priority objective of agricultural policy and is considered a fundamental instrument to sustain farmers' income. Either through the valorization of local products and the development of short supply channels in the most remote areas or in the concentration of the supply in cooperatives, it is in both cases a desirable element of that future vision.

 Finally, a comment on aspects included as desirable only in the vision of the future of the most mountainous area: digitization and diversification of activities. The first has become in these times of pandemic in a demanded aspect and considered as essential for the future of the rural world ^1^. Its non-inclusion in the vision of the more developed area is probably due to the fact that it already enjoys a high level of access to new technologies. Regarding the need for diversification of activities, including rural tourism, crafts, forestry sector or offering new services to the urban world, it arises significantly in areas where geographic and isolation conditions have not facilitated the development of a competitive agriculture.

Are all the source data and materials underlying the results available?

Yes

Is the study design appropriate and is the work technically sound?

Yes

Is the work clearly and accurately presented and does it engage with the current literature?

Yes

Are the conclusions drawn adequately supported by the results?

Yes

Are sufficient details of methods and analysis provided to allow replication by others?

Yes

Reviewer Expertise:

Agricultural economics and policy.

We confirm that we have read this submission and believe that we have an appropriate level of expertise to confirm that it is of an acceptable scientific standard.

## References

[1] : Long-Term Vision for Rural Areas: Contribution fromSHERPA science-society-policy platforms. *SHERPA Position Paper* .2021;

# Response

**Published:** 2021-05-14

**Authors:** Emilia Pellegrini

**Affiliations:** University of Bologna, Italy

Dear reviewers, thank you very much for your comments and reflections on the brief report. You raised many relevant issues for rural areas as well as for the difficulties of undertaking participatory approaches during the covid-19 era. We have addressed all your observations in the revised version of the brief report. Here, we discuss your observations in a more exhaustive way. Following up on your first observation, the gap we referred to was mainly interpreted as an economic gap. As highlighted by the OECD report on the policy implications of Covid-19 crisis for rural development (OECD, 2020), the economic gap between rural and metropolitan areas  - already widened by the 2008 financial crisis -  risks to get worst in the next years due to a much less diversified rural economy and to the demographic trends characterizing rural areas (i.e. ageing) that create a significant gap in terms of opportunities for development for these areas. Concerning your second observation, as you rightly pointed out, the wording “deep transformation” is probably exaggerated. While some transformations have undoubtedly occurred, we cannot infer that they will become permanent. To add a reflection on that, we can argue that the “depth” of such transformations will depend on how the resources derived from the incoming policies (e.g. CAP, Recovery Fund) will be dedicated to increasing the viability of rural areas and encouraging less energy-intensive agricultural systems.  As a recent study on Covid-19 and rural landscape in Italy has shown, hilly-mountainous rural landscapes– characterized by non-intensive and more diversified agriculture - exhibit on average 10% fewer cases of contagion compared to urban areas and areas with intensive agriculture (Agnoletti et al., 2020). The study concludes that the payoff that Italy could get from revitalizing non-intensive rural areas through EU funding sources can be very large because it would make the population more resilient to the current and future pandemics. Hence, transformations might become deep if more sustainable agriculture, digitalization, infrastructures, and services will be supported and, in turn, incremented in rural areas. Regarding your last remark, overall, there was a consensus among the participants to the MAP of the Emilia-Romagna region on the visions for rural areas. However, during the focus group with the MAP there was a disagreement on a sentence, included in the vision for hilly-mountainous rural areas, which stated that the care of the territory should be a priority over production. According to some experts, having a productive agriculture is fundamental to ensure the livelihood of rural areas and cannot come after the care of the territory. On the opposite, one stakeholder upheld this argument claiming that the future for hilly-mountainous areas will depend on the care of the territory that is also a trigger for a more productive agriculture, not vice versa. Likewise, all the improvements for rural areas of the future identified by MAP’s experts were confirmed by the respondents to the questionnaire except for the change “intensive and competitive agriculture dominated by big players” that in fact was not included in the vision.
